# Chikungunya Virus Infection and Diabetes Mellitus: A Double Negative Impact

**DOI:** 10.4269/ajtmh.16-0320

**Published:** 2016-12-07

**Authors:** Eddy Jean-Baptiste, Julia von Oettingen, Philippe Larco, Frédérica Raphaël, Nancy Charles Larco, Marie Marcelle Cauvin, René Charles

**Affiliations:** 1 FHADIMAC (Haitian Foundation of Diabetes and Cardiovascular Diseases), Port-au-Prince, Haiti; 2 Division of Pediatric Endocrinology, Montreal Children's Hospital, Montreal, Canada

## Abstract

The impact of chikungunya virus (CHIKV) infection on diabetic patients (DPs) has not been described. We aimed to compare clinical features of CHIKV infection in DPs and nondiabetic patients (NDPs), and to evaluate its effects on glycemic control among DPs. We recorded clinical information and, in DPs, glycemic control. Forty-six DPs and 53 NDPs aged ≥ 20 years living in Haiti, with acute CHIKV infection, were studied. Diabetes duration was 7.1 ± 6.1 years. The most common acute CHIKV clinical manifestations were arthralgia (100.0% DPs and 98.1% NDPs, *P* = 1.000) and fever (86.9% DPs and 90.5% NDPs, *P* = 0.750). In DPs as compared with NDPs, arthralgia was more intense (mean pain score of 6.0/10 ± 2.2 versus 5.1/10 ± 2.0, *P* = 0.04) and took longer to improve (8.2 ± 3.0 versus 3.5 ± 2.5 days, *P* < 0.0001). Severe arthralgia was more prevalent (58.7% versus 20.8%, *P* = 0.0002), as was myalgia (80.4% versus 50.9%, *P* = 0.003), and fever lasted longer (5.1 ± 1.8 versus 3.7 ± 1.7 days, *P* = 0.0002). Among DPs, median fasting capillary glucose before versus after disease onset was 132.5 and 167.5 mg/dL (*P* < 0.001), corresponding to a median increase of 26.8% (interquartile range: 14.4–50.1%). Antidiabetic medication was titrated up in 41.3%. In summary, among DPs, CHIKV infection has a significant negative impact on glycemic control and, compared with NDPs, results in greater morbidity. Close clinical and glycemic observation is recommended in DPs with CHIKV infection.

## Introduction

Chikungunya virus (CHIKV) infection is an arthropod-borne disease transmitted by *Aedes* mosquitoes, *Aedes aegypti* and *Aedes albopictus*. More than 75% of patients with CHIKV infection are symptomatic, most commonly presenting with fever and joint pains. Other possible symptoms include headaches, myalgia, arthritis, conjunctivitis, gastrointestinal and dermatological manifestations.^1^,^2^ Atypical presentations manifesting as neurological, ocular, cardiovascular, dermatological, renal, and other symptoms are possible.^3^,^4^ Patients with diabetes or other underlying medical conditions are thought to be at higher risk for severe disease.^5^ However, to our knowledge, there is no primary literature on the clinical manifestations and outcomes of CHIKV infection in patients with diabetes mellitus.

CHIKV was first isolated in 1952, during an outbreak in Tanzania from where it moved to cause many epidemics in Africa, India, Indian Ocean islands, and southern Asia, involving millions of people.^1^ The first outbreak in the Americas was reported in December 2013 from the French Caribbean island of Saint Martin.^6^ The isolated virus belongs to the Asian genotype, identified in late 1950s in the southern Asian countries, and is phylogenetically related to strains recently found in Indonesia, China, and the Philippines. The disease rapidly spread throughout most of the Caribbean islands as well as into Central America and Florida. In January 2014, autochthonous cases were reported from overseas French departments of Guadeloupe, Martinique, and French Guyana.^1^,^6^ In February 2014, the first confirmed cases were reported from the neighboring Dominican Republic.^7^

In Haiti, beginning in late March 2014, health-care providers noticed a rapidly increasing number of patients who presented with a virus-like syndrome but tested negative for acute dengue fever and malaria. On May 6, 2014, the Haitian Public Health Ministry confirmed the first 14 cases of CHIKV infection in the country. The illness rapidly spread reaching its peak in June 2014.^8^ In an outpatient internal medicine and endocrine clinic in the capital Port-au-Prince that is affiliated with Haitian Foundation of Diabetes and Cardiovascular Diseases (FHADIMAC), treating physicians observed high glycemic values in a large number of patients with diabetes who presented with a diagnosis of CHIKV infection. Most of these patients denied hyperglycemia, as determined by self-monitoring of blood glucose (SMBG), before CHIKV infection onset. They also seemed to more commonly complain of joint and muscle pain more prominently than patients without diabetes.

To our knowledge, beyond one case report on diabetic ketoacidosis in a patient with CHIKV infection,^9^ there are no published studies comparing glycemic control before and after the first clinical manifestation of this viral disease. Only one study that assessed the impact of diabetes on the clinical presentation of CHIKV infection suggested diabetes (with other comorbidities) as risk factor for hospitalization (2005–2006 outbreak of CHIKV fever on Reunion Island).^10^ However, the comparison between clinical features of hospitalized and nonhospitalized groups was not done, and reasons for hospitalization were not given.

Our study hypothesis was that CHIKV infection leads to substantial hyperglycemia among patients with diabetes and that disease severity was greater in patients compared with those without diabetes. We aimed to evaluate the effects of CHIKV infection on glycemic control among patients with diabetes, and to look for significant differences in the clinical presentation of the disease between patients with and without diabetes.

## Materials and Methods

**Figure 1.**
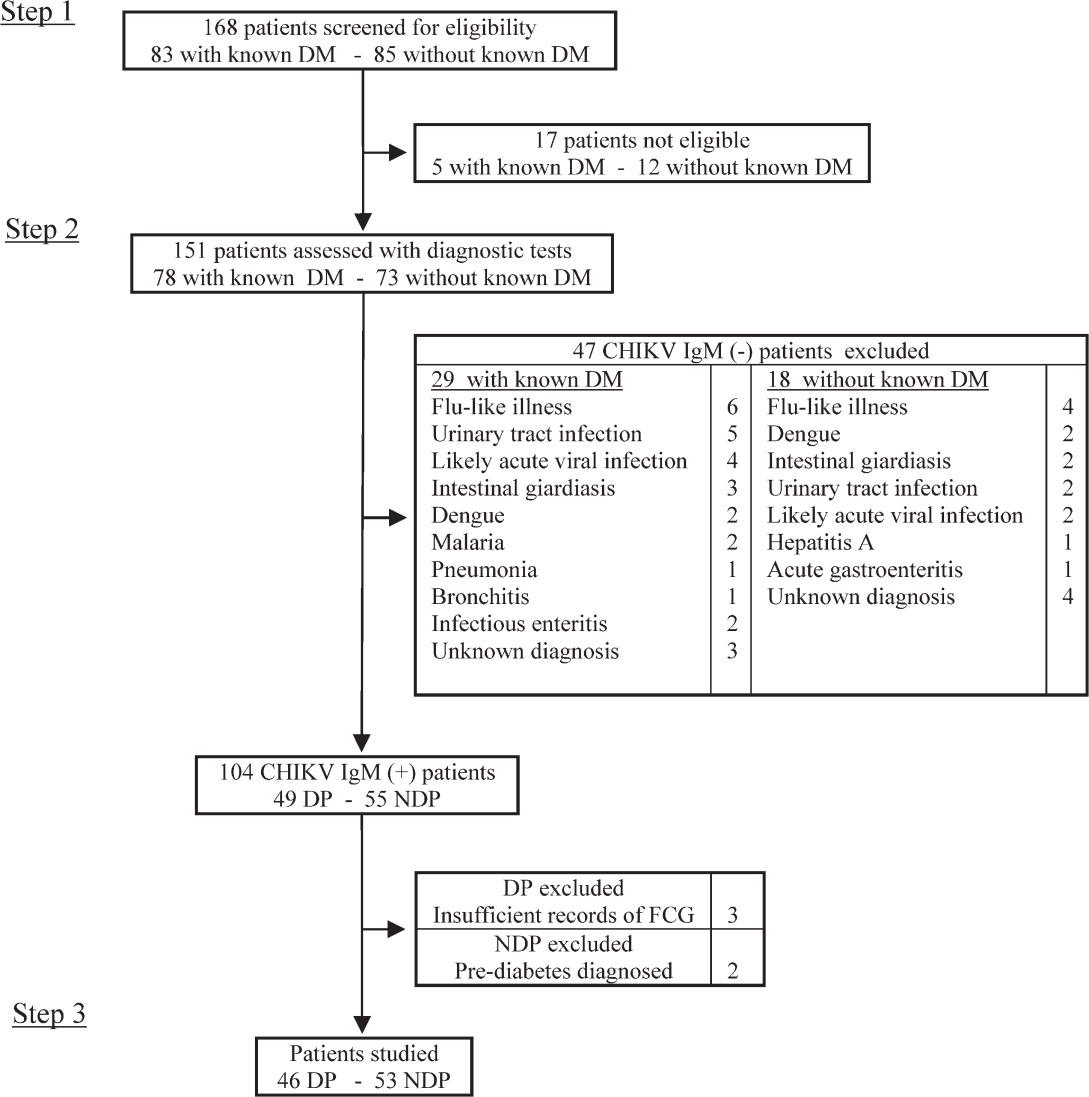
Study flowchart. CHIKV = chikungunya virus; DM = diabetes mellitus; DP = diabetic patient; FCG = fasting capillary glucose; NDP = nondiabetic patient.

The study was conducted from May 23, 2014 in an internal medicine and endocrinology outpatient clinic affiliated with FHADIMAC, and data collection lasted 9 weeks. Inclusion in the study arose from three steps (Figure 1

). First, all consecutive patients aged ≥ 20 years, with or without known diabetes, and with one or more signs and symptoms of acute fever, arthralgia/arthritis or periarticular edema, myalgia, headache, skin rash or pruritus, and acute fatigue were invited to participate in the study, and enrolled after giving verbal informed consent. Second, subjects reporting ≥ 2 of those symptoms underwent rapid diagnostic testing (RDT) for CHIKV infection (SD Bioline Chikungunya IgM, Standard Diagnostics, Yongin-si, South Korea), dengue fever (SD Bioline Dengue IgG/IgM, Standard Diagnostics) and *Plasmodium falciparum* malaria (SD Bioline Malaria Pf, Standard Diagnostics). The malaria RDT was performed at the first visit. The other two RDTs were carried out ≥ 6 days after disease symptom onset. Third, subjects with positive antichikungunya IgM and negative antidengue IgM as well as negative *P. falciparum* antigen were identified as having CHIKV infection only, and participated in the remaining study procedures.

Eligible patients were divided into two groups: diabetic patients (DPs), that is, patients with known diabetes and current CHIKV infection, and nondiabetic patients (NDPs), that is, patients without diabetes and with current CHIKV infection. To ensure absence of diabetes in the NDPs group, fasting capillary glucose (FCG) (Contour, blood glucose monitoring system, Bayer Health Care LLC, Japan) and point-of-care Hemoglobin A_1c_ (HbA_1c_) (DCA Vantage, Siemens Healthcare Diagnostics Inc., Tarrytown, NY) were performed on all patients without a history of diabetes, and patients were excluded if FCG or HbA_1c_ reached the prediabetes definition thresholds according to the American Diabetes Association^11^ (fasting blood glucose ≥ 100 mg/dL or HbA_1c_ ≥ 5.7%). DPs were excluded if they did not have a record of at least 2 FCG values within 7 days before disease onset and at least 2 FCG values before making any adjustment to their antidiabetic medication within the first 5 days after disease onset. The median FCG values were calculated before and after disease onset for each DP, and in the following text FCG in DPs refers to these median values. Among studied DPs, HbA_1c_ values before symptom onset were recorded. The study protocol was approved by the Haitian National Bioethics Committee.

A study physician administered a questionnaire to each participant to collect information on comorbidities, signs, and symptoms of the intercurrent illness with their duration, intensity of self-reported pain, and presence of debilitating status. Pain intensity or severity was assessed by using an 11-point numeric rating scale (NRS-11), with 0, 1–3, 4–6, and 7–10, representing no pain, mild pain, moderate pain, and severe pain, respectively. Pain was considered improved if the score determined at follow-up was at least two points lower than at diagnosis. Debilitating status was defined as the loss of ability to carry out daily tasks and capacity to live independently. We measured body mass index, blood pressure, and oral temperature according to standard methods. Treatment was provided based on clinical symptoms. The evolution of clinical manifestations in terms of duration and intensity was assessed during a follow-up visit or via a phone call performed by the study physician, 2–4 weeks after the first visit. In addition, the glycemic profiles of DPs were extracted from their medical files 1 month after disease onset.

Patient demographics and characteristics of both groups were assessed using univariate descriptive statistics. The χ^2^, Student's *t* test, and Kruskal–Wallis test, as appropriate, were used to assess significant differences in clinical presentation between the two groups.

Binomial logistic and multiple regression analysis with SPSS version 24 (IBM, Armonk, NY) was used to assess potential confounders in the difference of severity of clinical manifestations between both groups.

## Results

Of 168 patients screened, 99 (46 DPs and 53 NDPs) met eligibility criteria (Figure 1) and completed the study procedures. Baseline characteristics are presented in Table 1. Mean age was significantly higher (*P* = 0.043) for NDPs (53.2 years) when compared with DPs (47.4 years). There was no difference seen with regard to gender (56.5% and 56.6% of women among DPs and NDPs, respectively, *P* = 1.000). Eleven (23.9%) DPs and 11 (20.7%) NDPs were obese (*P* = 0.810). Mean duration of diabetes was 7.1 ± 6.1 years. Among DPs, HbA_1c_, measured 5.5 ± 4.2 weeks before disease onset, was 7.3 ± 1.1%, and median FCG during the 7 days before disease onset was 132.5 mg/dL (interquartile range [IQR]: 113.7–151.2). Two DPs (4.3%) were being treated with lifestyle modifications (LMs), 38 (82.6%) were taking oral antidiabetic agents (OAA), and six (13.0%) were using insulin. By definition of our inclusion criteria, no patient with CHIKV infection had concomitant positive dengue IgM test or *P. falciparum* malaria. Dengue IgG was positive in 12 (12.1%) of the 99 patients.

The clinical presentation is summarized in Table 2. Arthralgia and fever were the most common clinical manifestations in both groups. Arthralgia mainly affected large joints (ankles, knees, and wrists) and fingers (interphalangeal joints) in a symmetric pattern. It appeared more intense in DPs than in NDPs (average score of 6.0 ± 2.2 versus 5.1 ± 2.0, *P* = 0.040), and its severe form (score: 7–10), reported by 38 (38.3%) of all patients, was present in a significantly greater number of DPs compared with NDPs (27 [58.7%] DPs and 11 [20.8%] NDPs, *P* = 0.002). Arthralgia occurred before fever in 25 (54.3%) DPs and 22 (41.5%) NDPs (*P* = 0.230). Its improvement was reported after 8.2 ± 3.0 days versus 3.5 ± 2.5 days in DPs and NDPs, respectively (*P* < 0.0001). Fever resolved after 5.1 ± 1.8 days in DPs and 3.7 ± 1.7 days in NDPs (*P* = 0.0002). The triad of arthralgia, fever, and myalgia was found in 33 (71.7%) DPs and 23 (43.3%) NDPs (*P* = 0.008). Sixteen patients (11 DPs, 5 NDPs, *P* = 0.060) presented with a debilitating status with simultaneous presence of severe arthralgia and moderate to severe myalgia. Two DPs required hospitalization for 2 and 3 days for parenteral rehydration and insulin therapy adjustment. Myalgia always appeared a few hours after arthralgia or fever. Use of acetaminophen as analgesic or anti-inflammatory medicine after the onset of arthralgia, fever, or myalgia was not different between DPs and NDPs (78.2% versus 71.7%, *P* = 0.604). In a multivariable logistic regression model including diabetes, age, gender, and hypertension, only diabetes was identified as significant contributor to the presence of the triad of arthralgia, fever, and myalgia (*P* = 0.002), time to arthralgia improvement (*P* < 0.001), and fever duration (*P* = 0.002).

**Figure 2.**
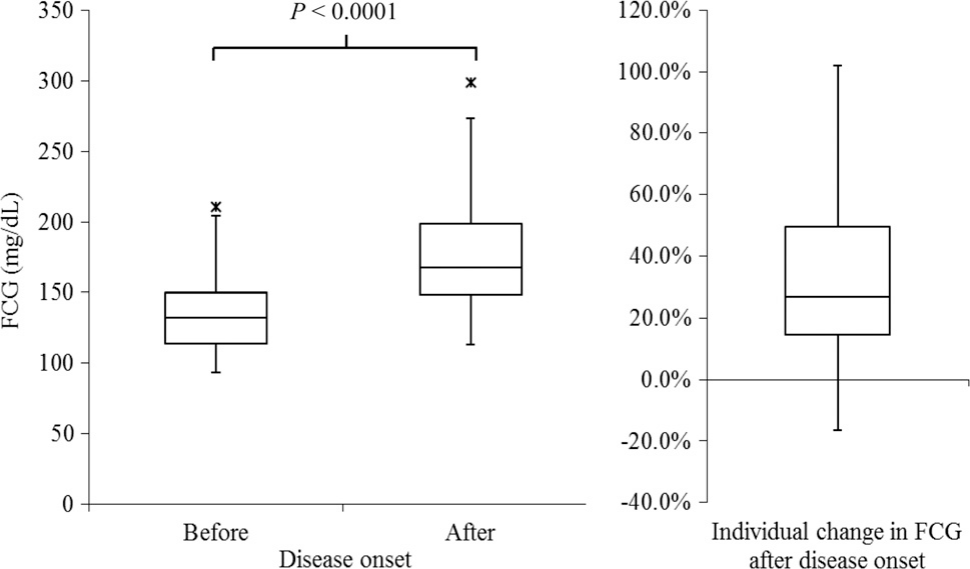
FCG before and after disease onset, and percentage of individual change in FCG after disease onset, among diabetic patients. The two asterisks indicate outliers > 1.5 interquartile range above the 75th percentile of all of the data. FCG = fasting capillary glucose.

Regarding changes in glycemic profile after illness onset, four (6.5%) DPs had a decrease in FCG going from 6.8% to 16.3% and 42 (93.5%) DPs had an increase in FCG (Figure 2

). For those with increased FCG, an increase of < 10%, 10–19%, 20–29%, and ≥ 30% was seen in two (4.7%), 12 (28.6%), six (14.3%), and 22 (52.4%) of patients, respectively. Overall, the median FCG before and after onset of disease among DPs was 132.5 mg/dL (IQR: 113.7–151.2) and 167.5 mg/dL (IQR: 147.0–201.0), respectively (*P* < 0.001), corresponding to a median increase of 26.8% (IQR: 14.4–50.1).

To achieve a fasting glycemia target of 80–130 mg/dL, antidiabetic medication was titrated up in 19 (41.3%) DPs. An insulin secretagogue was added to metformin in three patients. The two patients treated with LMs required addition of OAA. None of the DPs needed a downward adjustment of their antidiabetic therapy within 7 days after disease onset. Of the 42 DPs with increased FCG after illness onset, data from 37 medical files showed a decrease of glycemia levels from day 13 ± 2 in 26 DPs on OAA, and day 11 ± 3 in the six DPs initially on insulin (NS). Three DPs on OAA and the two DPs on LMs requiring OAA remained at the same range of glycemia.

## Discussion

To our knowledge, this is the first study comparing clinical features of CHIKV infection among patients with and without diabetes and that determines the impact of the disease on glycemic control among DPs. Our results showed a double negative impact of this infection in patients with diabetes. First, DPs, as compared with NDPs, presented with more intense arthralgia and higher prevalence of myalgia, as well as longer time to improvement of arthralgia and longer duration of fever. The combination of arthralgia, fever, and myalgia was more prevalent among DPs compared with NDPs. In addition, CHIKV infection adversely impacted glycemic control in the DPs group, leading to significant up-titration of antidiabetic medication in more than 40% of DPs.

Although the epidemiological evidence of the effects of arboviral infections on diabetes remains limited, other studies showed that diabetes increased risk for severe clinical presentation of two other important arboviruses, dengue fever and West Nile virus disease.^12^–^14^ Our findings regarding the negative impact of diabetes on the clinical manifestations of CHIKV infection are in line with these studies.

The most common clinical manifestations in our study were overall similar to those reported in prior literature.^1^ Arthralgia was the predominant symptom, involving almost all patients; fever was present in 85% and myalgia was reported by more than half of patients. However, in contrast to symptom chronology reported in prior literature,^1^,^2^ arthralgia appeared before rather than after fever in more than 40% of patients of both groups. We have no definite explanation for this difference in presentation. Because there was a 6-day time limit to perform the diagnostic test of CHIKV infection after symptom onset, retrospective symptom self-report was used (6.5 and 7.0 days on average after illness onset among DPs and NDPs), potentially limiting accuracy of timing of reported arthralgia and fever. However, this finding was similar in both groups. The use of acetaminophen, as it started after arthralgia or fever onset, could not explain this feature. Furthermore, factors related to the virus strain itself or to the host response in our population could have played a role in this symptom chronology. To our knowledge, the strain implicated in the Haitian outbreak has not been studied regarding its RNA genome and its degree of similarity to Caribbean sequences.

Skin rash is usually observed in 20–80% of CHIKV infection cases.^4^ Its rate of 19.2% in our study might be an underestimate, possibly due to increased difficulty in identifying skin eruptions in dark-skinned persons. The presence of pruritus in half of our patients may indicate that the actual rate of skin involvement was higher than estimated by skin rash alone.

Biopsy specimen from CHIKV-infected humans have suggested a specific cell and tissue tropism of CHIKV for fibroblasts in joint, muscle, and skin, possibly explaining CHIKV's predominant clinical manifestations in these tissues.^15^ Increased prevalence of myalgia, greater severity of arthralgia, and longer duration of fever in DPs versus NDPs resulted in higher morbidity in DPs. As a consequence, it is not surprising that the illness also tended to be more disabling in DPs. The pathophysiology leading to an increase in disease severity in DPs versus NDPs is unknown. An interesting current hypothesis is a defect or malfunction of the type I interferon pathway in patients with diabetes, causing a decrease in CHIKV clearance and, as a result, increased viral loads and greater disease severity.^15^–^17^ On the other hand, age and hypertension did not appear to be contributing factors to increased morbidity among DPs in this study. The adverse effect of CHIKV infection on glycemic control was striking. It is noteworthy that none of the DP had taken steroids or anti-inflammatory medicines. In addition to insulin resistance related to most of infectious intercurrent illnesses, CHIKV infection appears to have an additional effect on glycemic levels in DPs, as judged by the magnitude of the hyperglycemic effect. A recent proteomics analysis of CHIKV infection in vitro demonstrated that among 45 downregulated proteins in cells infected with CHIKV, proteins involved in carbohydrate and lipid metabolism predominated,^18^ possibly indicating that CHIKV may have an inherent effect on glucose metabolism that particularly affects DPs. Obviously, more investigations are needed to find out the explanations of the deleterious correlation between diabetes and CHIKV infection.

The clinical features in all patients studied and the effects of CHIKV infection on glycemic control in DPs are likely to be directly related to their CHIKV infection. We minimized confounding of clinical features by ensuring the absence of coinfection with *P. falciparum* malaria and dengue fever, two important endemic infectious diseases that share nonspecific clinical manifestations with CHIKV infection. SD Bioline Malaria Ag Pf is specific to *P. falciparum*, and belongs to the RDTs that have the highest malaria detection rate. According to the World Health Organization, its panel detection score is > 95% for very low parasite density (50–100 parasites/μL).^19^ Its high performance was confirmed in Haiti in 2010.^20^ SD Bioline Dengue IgG/IgM showed a low diagnostic performance in some evaluation studies.^21^,^22^ However, none of the patients with CHIKV infection showed clinical manifestations suggestive of acute dengue fever at presentation or follow-up. Further, the association of acute fever and arthralgia, which was present in 87.8% of participants, has been found to be highly predictive of CHIKV infection in previous studies, and the debilitating polyarthralgia has had a positive predictive value of ≥ 80% for CHIKV infection in outbreak areas.^23^

Our study has limitations. We did not take into account asymptomatic infections, which can vary from 3% to 25% according to the involved strains and the outbreaks.^2^ However, under the assumption that asymptomatic infections are more likely to occur in otherwise healthy persons, this may, if anything, have biased our results toward a less than actually detected difference in presentation between DPs and NDPs. Further, despite best attempts at ruling out concurrent infections with dengue virus, we cannot exclude the possibility that some of the 12 patients with dengue IgG-positive result were also suffering from secondary acute dengue infection. The latter predominates in dengue-endemic settings and can generate production of IgG antibodies within the first days of symptom onset (anamnestic response), while the blood levels of the IgM antibodies are not yet detectable by RDTs.^22^,^24^ We were limited to a relatively small number of SMBG values to obtain an approximate estimation of the glycemic profile before and after illness onset due to the short mean duration of the clinical manifestations of CHIKV infection and our clinic's protocol which recommends adjustment of antidiabetic agent dose after two hyperglycemic readings or immediately after major hyperglycemia. Despite this small number of values, the difference between glycemic profiles before and after symptom onset was statistically significant and appeared clinically relevant. We were unable to include viral load and cytokine profiles in our study as these are not currently available in Haiti. This may have prevented us from finding further biological data in both patient groups to support the hypotheses explaining the causal relationship between diabetes and severe clinical presentation of CHIKV infection. At the follow-up evaluation, only fever and arthralgia appeared reasonably accurate measures of intensity and exact duration of symptoms. Consequently, we could not make a complete comparison between both groups on symptom evolution. Finally, the small sample size may not have represented the general population, and our results need to be confirmed by larger studies.

In summary, this is the first study to show that CHIKV infection has a significant negative impact on glycemic control among adult patients with diabetes and causes greater morbidity among DPs compared with NDPs. Close clinical observation, high SMBG frequency, and prompt adjustment of diabetes treatment regimen are recommended in patients with diabetes suffering from CHIKV infection.

## Figures and Tables

**Table 1:** Baseline characteristics of patients

	DPs (*N* = 46)	NDPs (*N* = 53)
Delay between symptom onset and first visit (days)	5.1 ± 1.9	5.7 ± 2.6
Delay between symptom onset and enrollment (days)	6.5 ± 0.8	7.0 ± 1.5
Delay between first visit and follow-up (days)	21.8 ± 3.7	22.4 ± 4.0
Age (years)*	47.4 ± 15.6	53.2 ± 12.1
Female sex, *n* (%)	26 (56.5)	30 (56.6)
BMI (kg/m^2^)	28.1 ± 4.7	26.4 ± 5.3
BMI ≥ 30 kg/m^2^, *n* (%)	11 (23.9)	11 (20.7)
Systolic blood pressure (mmHg)	135.8 ± 25.3	129.2 ± 25.4
Diastolic blood pressure (mmHg)	83.8 ± 11.4	81.5 ± 13.2
Temperature (°C)	36.8 ± 0.5	36.2 ± 4.7
Duration of diabetes (years)	7.1 ± 6.1	–
HbA_1c_ (%)†	7.3 ± 1.1	5.4 ± 0.2
Median FCG (mg/dL)‡	132.5 (113.7–151.2)	86 (79–89.8)
Diabetes treatment, *n* (%)		
Lifestyle modifications only	2 (4.3)	–
Oral medication	38 (82.6)	–
Insulin	6 (13.0)	–
Comorbidities		
Hypertension§	17 (36.9)	14 (26.4)
Cardiovascular disease¶	2 (0.04)	1 (0.01)
Asthma	1 (0.02)	3 (0.5)
Sickle cell anemia	0 (0.0)	1 (0.01)

BMI = body mass index; DPs = diabetic patients; FCG = fasting capillary glucose; NDPs = nondiabetic patients; SD = standard deviation. Data are mean ± SD, *n* (%), or median (interquartile range).

* *P* < 0.05.

† Measured 5.5 ± 4.2 weeks before disease onset for DPs and at first visit for NDPs.

‡ Median FCG within 7 days before disease onset for DPs, and median FCG at first visit for NDPs.

§ Defined as previous diagnosis of hypertension by a physician, and including persons being treated for hypertension. *P* = 0.13.

¶ Including history of coronary artery disease, congestive heart failure, heart attack, or stroke.

**Table 2:** Clinical presentation of patients

	Diabetic patients (*N* = 46)	Nondiabetic patients (*N* = 53)	*P* value
Arthralgia	46 (100)	52 (98.1)	1.000
Score of arthralgia*	6.0 ± 2.2	5.1 ± 2.0	0.040
Severe arthralgia†	27 (58.7)	11 (20.7)	0.0002
Score of severe arthralgia	8.2 ± 0.7	7.5 ± 0.8	0.021
Occurrence of arthralgia before fever	25 (54.3)	22 (41.5)	0.230
Days before arthralgia improvement	8.2 ± 3.0	3.5 ± 2.5	< 0.0001
Fever	40 (86.9)	48 (90.5)	0.750
Number of days with fever	5.1 ± 1.8	3.7 ± 1.7	0.0002
Myalgia	37 (80.4)	27 (50.9)	0.003
Score of myalgia*	5.4 ± 2.2	4.6 ± 2.5	0.182
Arthralgia + fever + myalgia	33 (71.7)	23 (43.3)	0.008
Debilitating illness‡	11 (23.9)	5 (9.4)	0.060
Chills	25 (54.3)	28 (52.8)	1.000
Pruritus	27 (58.7)	24 (45.2)	0.277
Fatigue	23 (52.3)	26 (49.0)	1.000
Back pain	26 (56.5)	21 (39.6)	0.109
Difficult walking	25 (54.3)	22 (41.5)	0.230
Headache	15 (32.6)	25 (47.1)	0.156
Lymphadenopathy	21 (45.7)	16 (30.1)	0.145
Joint swelling	17 (36.9)	19 (35.8)	1.000
Rash	10 (21.7)	9 (16.9)	0.614
Use of acetaminophen	36 (78.2)	38 (71.7)	0.604

SD = standard deviation. Data are *n* (%) or mean ± SD.

* Assessed with an 11-point numeric rating scale from 0 (no pain) to 10 (maximal pain).

† Score of 7–10.

‡ With loss of the ability to carry out daily tasks and the capacity to live independently.
